# Epigenetic memory gained by priming with osteogenic induction medium improves osteogenesis and other properties of mesenchymal stem cells

**DOI:** 10.1038/srep11056

**Published:** 2015-06-08

**Authors:** Yunfeng Rui, Liangliang Xu, Rui Chen, Ting Zhang, Sien Lin, Yonghui Hou, Yang Liu, Fanbiao Meng, Zhenqing Liu, Ming Ni, Kam Sze Tsang, Fuyuan Yang, Chen Wang, Hsiao Chang Chan, Xiaohua Jiang, Gang Li

**Affiliations:** 1 Department of Orthopaedics, Zhongda Hospital, Southeast University, 87 Ding Jia Qiao, Nanjing 210009, Jiangsu, PR China; 2 Department of Orthopaedics & Traumatology, The Chinese University of Hong Kong, Prince of Wales Hospital, Hong Kong SAR, PR China; 3 Epithelial Cell Biology Research Center, School of Biomedical Sciences, Faculty of Medicine, The Chinese University of Hong Kong, Hong Kong SAR, PR China; 4 Department of Anatomical and Cellular Pathology, The Chinese University of Hong Kong, Hong Kong SAR, PR China; 5 Lui Che Woo Institute of Innovative Medicine, Faculty of Medicine, The Chinese University of Hong Kong, Hong Kong SAR, China; 6 Stem Cells and Regenerative Medicine Laboratory, Li Ka Shing Institute of Health Sciences, The Chinese University of Hong Kong, Hong Kong SAR, China; 7 Key Laboratory for Regenerative Medicine, Ministry of Education, School of Biomedical Sciences, The Chinese University of Hong Kong, Hong Kong SAR, PR China; 8 The Chinese University of Hong Kong, Shenzhen Research Institute, Shenzhen, PR China; 9 The Department of Orthopaedics, The General Hospital of Chinese People’s Liberation Army, Beijing, PR China

## Abstract

Mesenchymal stem cells (MSCs) are highly plastic cells that are able to transdifferentiate or dedifferentiate under appropriate conditions. In the present study, we reported here that after *in vitro* induction of osteogenic differentiation, MSCs could be reverted to a primitive stem cell population (dedifferentiated osteogenic MSCs, De-Os-MSCs) with improved cell survival, colony formation, osteogenic potential, migratory capacity and increased expression of Nanog, Oct4 and Sox2. Most importantly, our results showed great superiority of the De-Os-MSCs over untreated MSCs in ectopic bone formation in vivo. Furthermore, Nanog-knockdown in MSCs could reverse these enhanced properties in De-Os-MSCs in vitro, indicating a central role of Nanog in the transcriptional network. In addition, epigenetic regulations including DNA methylation and histone modifications may play important roles in regulating the de-osteogenic differentiation process. And we found decreased methylation and promoter accrual of activating histone marks, such as H3K4me3 and H4ac on both Nanog and Oct4 gene promoters. Taken together, our study demonstrated that epigenetic memory in De-Os-MSCs gained by priming with osteogenic induction medium favored their differentiation along osteoblastic lineage with improved cell survival and migratory abilities, which may have application potential in enhancing their regenerative capacity in mammals.

Bone possesses the intrinsic regeneration capacity as part of the repair process in response to injury, during skeletal development or continuous remodeling throughout adult life^1^. However, some complex clinical conditions, such as large bone defects or atrophic non-unions and osteoporosis, require bone regeneration in too large quantity, and tissue engineering approach was developed to favor the regeneration of a new functional tissue^2^. Several characteristics of mesenchymal stem cells (MSCs), such as the potential to differentiate into multiple lineages and the ability to be easily expanded ex vivo while retaining their original lineage differentiation commitment, make these cells very promising targets for therapeutic use in regenerative medicine and tissue engineering^3^. However, the harsh ischemic and cytokine-rich microenvironment in the bone fracture site, infiltrated by the inflammatory and immune cells, offers a significant challenge to the transplanted donor stem cells. Low cell survival rate and differentiation in vivo after MSCs transplantation has significantly hindered the effectiveness of stem cell therapy^4^,^5^,^6^,^7^. Of note, MSCs are extremely plastic in that they can cross oligo-lineage boundary and transdifferentiate into cells of unrelated lineages, including neurons, hepatocytes and epithelial-like cells under specific conditions^8^,^9^,^10^,^11^. Interestingly, recent studies from both our group and others have demonstrated that dedifferentiation is a prerequisite for MSCs to change their cell fate and redifferentiate into a different linage^12^,^13^. Furthermore, our recent study demonstrated that MSCs could be reprogrammed in vitro via neuronal differentiation and dedifferentiation with enhanced therapeutic efficacy in a rat model with ischemic brain damage^14^. This is of particular interest, since the finding provides a potential approach to overcome some of the major hurdles faced by current MSC-based therapy.

While the application of transdifferentiation and dedifferentiation as potential therapeutic strategies has attracted much attention in MSC-based therapy, the molecular mechanisms underlying MSCs plasticity are largely unknown. It has been suggested that the plastic capacity of MSCs could be explained by their complex transcriptome, which encodes a wide range of proteins involved in different developmental pathways and in a large number of diverse biological processes^15^. To this end, a large body of studies has been focused on identifying a number of extrinsic regulators and their intrinsic target transcription factors that control MSCs plasticity^16^,^17^. Nevertheless, accumulating evidence indicates that stem cell fate and function is determined by DNA-binding transcription factors that are regulated more specifically at the epigenetic level as we learned from pluripotent stem cells, such as embryonic stem (ES) cells and induced-pluripotent stem cells (iPSC)^18^,^19^,^20^. It has been proposed that, although with conflicting results, the pluripotency marker genes, including Nanog, Sox2 and Oct4, play a similar role in adult stem cells. But the exact molecular mechanisms regulating the undifferentiated state of MSCs are rarely known, and the roles of these three pluripotency marker genes in MSCs are not fully revealed. For example, people have tried to improve the stemness of MSCs by overexpressing Oct4 and Nanog, and found that Oct4 and Nanog indeed could promote cell proliferation, colony formation and chondrogenesis of MSCs, but showed converse effects on adipogenesis^21^. Masahiro *et al.* have also found that overexpression of Sox2 or Nanog can promote the osteogenesis of human MSCs as well as maintain their expansion^22^. Thus, while epigenetic regulatory mechanisms that govern MSCs plasticity remain largely elusive, they are the crucial missing pieces linking extracellular stimuli to transcriptional regulation and downstream intracellular signaling leading to MSCs maintenance or lineage commitment^23^.

Up to now, DNA methylation and histone modifications are the most important epigenetic regulations which possess the power to control the differentiation or maintain the self-renewal of MSCs^24^. It is known that changes in the methylation states of the CpG islands in the promoter regions or the first exon are inversely correlated with the expressions of the corresponding genes. Histone modifications can influence the interactions of transcription factors with chromatin. The analysis of histone modifications in embryonic stem cells has found many bivalent loci that are associated with both H3 lysine 27 trimethylation (H3K27me3) and H3 lysine 4 trimethylation (H3K4me3)^25^,^26^,^27^,^28^. The bivalent loci in MSCs are often low in DNA methylation and can be further methylated or activated, which are distinct from those in the embryonic stem cells and differentiated cells^29^. Targeted DNA methylation within the Trip10 promoter has been shown to accelerate the MSCs to neuron or osteocyte differentiation^30^.

In this study, following our previous study, we asked whether osteogenic-MSCs could undergo dedifferentiation as well as the previously demonstrated neurogenic-MSCs, and if yes, what might be the epigenetic mechanisms underlying this process. The results showed that epigenetic memory in De-Os-MSCs gained by priming with osteogenic induction medium favored their differentiation along osteoblastic lineage with improved cell survival and migratory abilities. In addition, we demonstrated that Nanog played a critical role in maintaining the dedifferentiation phenotype. Epigenetic mechanisms involving both DNA methylation and histone modification contribute to opening of the chromatin structure and activation of pluripotent genes during dedifferentiated-mediated reprogramming.

## Results

### De-differentiation of MSCs-derived osteogenic progenitors

**Figure 1:**
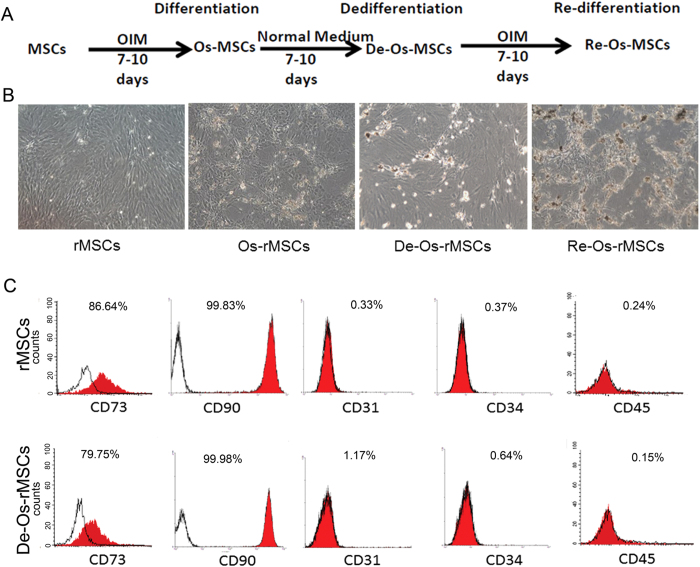
De-differentiation of MSCs-derived osteogenic progenitors. (**A**) Schematic diagram illustrating the procedure for deriving De-Os-MSCs. The rMSCs that went through osteogenic differentiation, dedifferentiation and redifferentiation were illustrated. (**B**) Representative phase contrast image showing rMSCs were induced to undergo osteogenic differentiation, dedifferentiation and redifferentiation. **(C)** Cell surface markers of rMSCs and De-Os-rMSCs. Antibodies against CD90, CD73, CD31, CD34 and CD45 were used to characterized rMSCs and De-Os-rMSCs.

We have previously reported that after in vitro neuronal differentiation and dedifferentiation, rat bone marrow derived-MSCs (rMSCs) exhibited enhanced cell survival and neuronal differentiation potential both in vitro and in vivo^14^. This finding promoted us to explore whether the dedifferentiation process is lineage specific. The general process of osteogenic differentiation and dedifferentiation was schematically illustrated in Fig. 1A. After 7–10 days of osteogenic differentiation, MSCs changed from a fibroblastic appearance to a more polygonal appearance and started to form nodules. After 14 days of differentiation, multiple nodules were formed in the culture (Fig. 1B, Os-rMSCs). These early committed cells were then cultured under basal media that support stem cell growth. Withdrawal of OIM reverted MSC-derived osteogenic progenitors to characteristic mesenchymal morphology after incubation in basal media for another 7–14 days (Fig. 1B, De-Os-rMSCs). Then, we compared the cell-surface antigen profiles of De-Os-rMSCs to those of undifferentiated counterparts. The fluorescence-activated cell sorting (FACS) profiling showed that rMSCs expressed CD90 (99.83%), CD73 (86.64%), but were negative for CD31 (0.33%), CD34 (0.37%) and CD45 (0.24%), and the De-Os-rMSCs retained their immunophenotype similar to that of undifferentiated rMSCs (Fig. 1C).

### Osteogenic advantage of De-Os-MSCs in vitro

**Figure 2:**
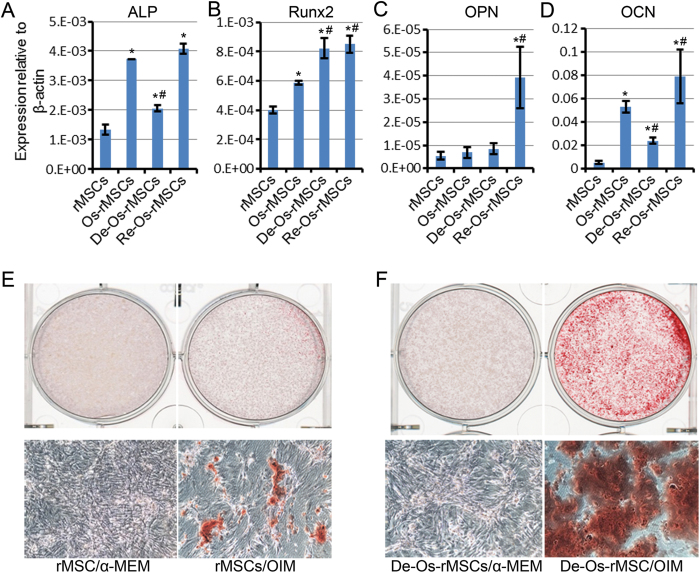
De-Os-MSCs showed osteogenic advantage in vitro. (**A**) Total RNA were extracted from rMSCs, Os-rMSCs, De-Os-rMSCs and Re-Os-rMSCs. The relative expression levels of ALP, Runx2, OPN and OCN were checked by qRT-PCR. β-actin was used as an internal control. The data are expressed as mean ± SD (n = 3), *p < 0.05 compared to MSCs, #p < 0.05 compared to Os-MSCs. **(B)** Alizarin Red S staining of calcium deposits formed by MSCs. The untreated rMSCs and De-Os-rMSCs were cultured in α-MEM or osteogenic induction medium for 10 days, then the cells were fixed and stained with Alizarin Red S.

We next tested the in vitro osteogenic differentiation capacity of De-Os-MSCs in comparison with untreated MSCs. The mRNA expression levels of genes related to osteogenesis were detected by qRT-PCR. As shown in Fig. 2A–D, the expression of ALP (alkaline phosphatase) and Runx2 (runt-related transcription factor 2), which are early markers for osteogenic commitment, was markedly increased in De-Os-rMSCs compared with undifferentiated rMSCs, as well as the late osteogenic marker OCN (Osteocalcin). And the expression levels of OPN (Osteopontin) and OCN were dramatically increased in Re-Os-rMSCs (Fig. 2C,D). To confirm the osteogenic commitment of rMSCs and De-Os-rMSCs, Alizarin Red S staining was used to detect the formation of calcium deposit. The results showed that after 10 days of OIM induction, mineralization was seen in both De-Os-rMSCs and untreated rMSCs upon osteogenic induction. However, there were significantly more Alizarin Red S-positive calcium nodules formed in the De-Os-rMSCs group (Fig. 2E,F). These data, together with our Alizarin Red S staining showing much enhanced calcium deposition in the Re-Os-rMSCs, indicating that De-Os-rMSCs retains osteogenic traits, and therefore, possesses higher potential for redifferentiation into osteoblasts.

### Other properties of De-Os-rMSCs

Then we determined whether there were changes in adipogenic and chondrogenic differentiation potential after induction of osteogenic differentiation and dedifferentiation. Our result showed that De-Os-rMSCs had lower adipogenic differentiation as demonstrated by Oil Red O staining (Supplementary Figure 1A), and also lower chondrogenic differentiation as demonstrated by Alcian Blue staining (Supplementary Figure 1B).

**Figure 3:**
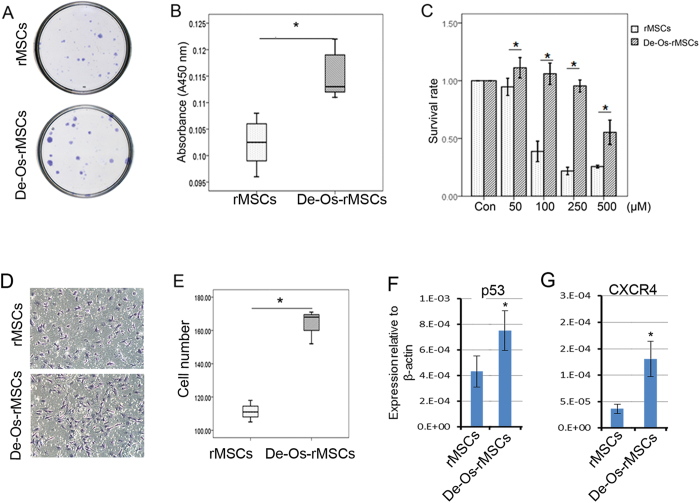
De-Os-rMSCs exhibited advantages in clonogenicity, proliferation, cell survival and migration. (**A**) Clongenic assay. The untreated rMSCs and De-Os-rMSCs were plated at 100 cells per 20 cm^2^ dish and cultured for 10 days, respectively. The cells were then stained with 0.5% crystal violet to view the colonies. (**B**) Cell proliferation assay. The rMSCs and De-Os-rMSCs were plated at 2,000 cells/well in normal medium in a 96-well plate and incubated at 37 °C with 5% CO_2_. At day 3, cell proliferation was assessed using the BrdU assay as described in Materials and Methods. The data are expressed as mean ± SD (n = 3), *p < 0.05. (**C**) De-Os-rMSCs exhibited survival advantage over untreated rMSCs. The untreated rMSCs and De-Os-rMSCs were plated in 96-well plates. At days 3, these cells were challenged with 0–500 μM H_2_O_2_ for 12 hours. Then, the cells were incubated with Alamar Blue for 2.5 hours at 37 °C. The metabolic rate of the cells was determined at 570 nm, with reference wavelength at 600 nm. Values are expressed as mean ± SD of three independent experiments. *p < 0.05. (**D**&**E**) De-Os-rMSCs exhibited advantage in migration over untreated rMSCs. An equal number of untreated rMSCs and De-Os-rMSCs suspended in α-MEM were added into the upper layer of BD Falcon cell culture insert, respectively, and the rMSCs migrated through the membrane were detected with crystal violet staining. The number of MSCs that passed through the membrane was counted (n = 3). Values are expressed as mean ± SD of three independent experiments. *p < 0.05. (**F**&**G**) Total RNA were extracted from rMSCs and De-Os-rMSCs. The relative expression levels of p53 and CXCR4 were checked by qRT-PCR. β-actin was used as an internal control. The data are expressed as mean ± SD (n = 3), *p < 0.05.

To determine whether De-Os-rMSCs exhibit any difference in stem cell potency, we evaluated the colony forming ability, cell proliferation, cell survival and cell migration in naïve rMSCs and De-Os-rMSCs. Our results showed that De-Os-rMSCs formed more and larger colonies as compared to untreated rMSCs Fig. 3A). This could be related to the increase in cell proliferation of De-Os-rMSCs, as demonstrated by Brdu assay (Fig. 3B). In addition, De-Os-rMSCs exhibited a survival advantage over untreated rMSCs under the circumstance of oxidative stress. As shown in Fig. 3C, De-Os-rMSCs had higher cell survival capacity compared to untreated rMSCs when both of them were challenged by H_2_O_2_ at different concentrations for 12 hours. As revealed by transwell migration assay, the migratory capability was also significantly enhanced in De-Os-rMSCs compared to untreated rMSCs (Fig. 3D,E). In addition, we also checked the expression levels of some genes that were known to be involved in cell proliferation and migration by qRT-PCR, and the result showed that p53 and CXCR4 were significantly increased in De-Os-rMSCs (Fig. 3F,G), implying that upregulation of p53 and CXCR4 might account for the increased proliferation and migration showed by De-Os-rMSCs.

### Osteogenic differentiation advantage of De-Os-rMSCs in vivo

**Figure 4:**
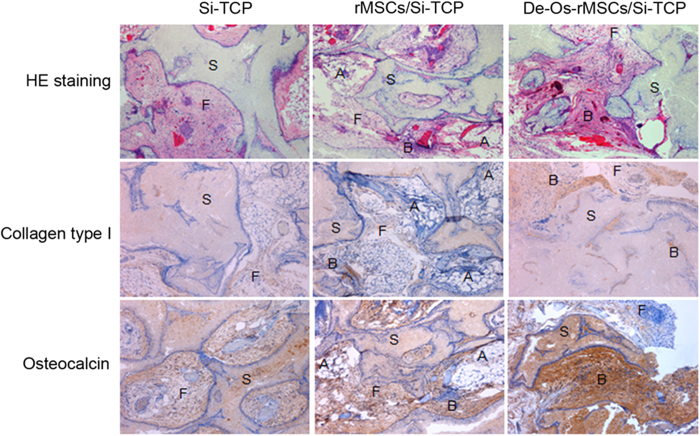
De-Os-rMSCs formed more ectopic bone in nude mice. The untreated rMSCs and De-Os-rMSCs were loaded onto sterilized porous calcium phosphate restorable granules, and then implanted subcutaneously into the dorsal surfaces of nude mice. The transplants were harvested 8 weeks later for histological examination. The sections were stained with routine hematoxylin and eosin, and Immunohistochemistrical staining with anti-collagen type I or anti-OCN antibody. A: adipose tissue; F: fibrous tissue; S: Si-TCP biomaterial remnants; B: bone tissue.

To further evaluate the advantages of De-Os-rMSCs in osteogenic differentiation in vivo, De-Os-rMSCs were loaded onto sterilized Skelite® resorbable Si-TCP bone graft substitutes and implanted subcutaneously at the dorsal sides of nude mice. The transplants were harvested 8 weeks later and subjected to histological examination with HE staining or immunohistochemical analysis to detect the distribution of osteoid, and expression of collagen type I and OCN. Our results showed that the transplantation of De-Os-rMSCs with Si-TCP resulted in more bone-like tissue formation, less loose fibrous tissue and adipose tissue formation around the scaffold compared to the untreated rMSCs with Si-TCP in nude mice (Fig. 4). The formation of bone-like tissue was confirmed by the presence of typical collagen birefringence of bone tissue and the high expression of collagen type I and osteocalcin. These results indicated that De-Os-rMSCs were superior to untreated rMSCs in ectopic bone formation in vivo.

### Epigenetic regulation of Nanog and Oct4 in De-Os-rMSCs

**Figure 5:**
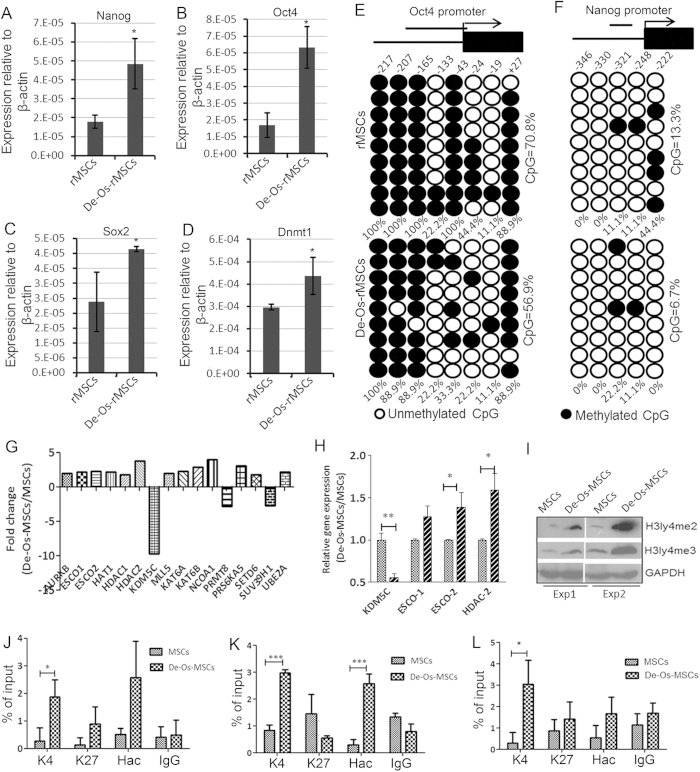
Epigenetic regulation of Nanog and Oct4 in De-Os-rMSCs. (**A**-**D**) Total RNA were extracted from rMSCs and De-Os-rMSCs. The relative expression levels of Oct4, Nanog, Sox2 and Dnmt1 were checked by qRT-PCR. β-actin was used as an internal control. The data are expressed as mean ± SD (n = 3). *p < 0.05. (**E**-**F**) DNA methylation status of Oct4 and Nanog promoters in rMSCs and De-Os-rMSCs using sodium bisulfite sequencing. The top panel indicates the CpG dinucleotide position of the Oct4 and Nanog promoter regions and the numbers show positions of CpGs relative to the translation start site. Each PCR product was subcloned and subjected to nucleotide sequencing analysis. Nine representative sequenced clones were depicted by filled (methylated) and open (unmethylated) circles for each CpG site. (**G**-**L**) Chromatin configurations in De-Os-rMSCs compared to rMSCs. (**G**) Differentially expressed histone modifying enzymes in De-Os-MSCs and MSCs examined by focused PCR array. (**H**) QRT-PCR analysis confirmed the differentially expressed histone modifying enzymes as identified in (**G**). (**I**) Western blot analysis showing the expression of H3K4me3 and H3K4me2 were increased in De-Os-MSCs compared to MSCs. The data showing here are two independent experiments. (**J**-**L**) Increased occupany of H3K4me3 and H4ac on Oct4 (primer1 and primer 2 as detailed in Supplementary Table 2) and Nanog promoters. The histone modifications of Oct4 (**J**&**K**) and Nanog (**L**) promoters were analyzed by CHIP-PCR assay. CHIP was done using anti-H3K4me3, anit-H3K27 and anti-H4ac monoclonal antibodies, and PCR was performed with primers listed in supplementary table. Normal rabbit IgG was used as a negative control. Values are expressed as mean ± SD of three independent experiments. *p < 0.05.

Next, we asked what were the molecular mechanisms underlying the enhanced stem cell potency in De-Os-MSCs. Oct4, Sox2 and Nanog are three major pluripotent genes critical for the maintenance of ESCs pluripotency and reprogramming of iPSCs^31^. Interestingly, our qRT- PCR demonstrated significantly higher expression of Nanog, Oct4 and Sox2 in De-Os-rMSCs compared with that in untreated rMSCs, documenting a more primitive phenotype of these cells (Fig. 5A–C). Since Dnmt1 but not Dnmt3 has been proved to be involved in the Oct4 and Nanog-mediated maintenance of stem cell properties in MSCs^32^, we also checked the expression level of Dnmt1 in De-Os-rMSCs. The qRT-PCR result showed that Dnmt1 was significantly increased in De-Os-rMSCs (Fig. 5D), meaning that Dnmt1 might play an important role in maintaining self-renewal state in De-Os-rMSCs. The methylation status of DNA is the most common epigenetic modification of the genome in mammalian cells^13^. As the first step to explore probable epigenetic mechanisms involved in upregulating Nanog and Oct4, we calculated the percentage of methylated CpG loci (percent CpG methylation) in the total eight CpG loci in Oct4 promoter and five CpG loci in Nanog promoter, separately. We found that Oct4 promoter was hypermethylated whereas Nanog promoter is hypomethylated in rMSCs (70.8% and 13.3% CpG methylation) (Fig. 5E,F). In De-Os-rMSCs, the methylation status of Nanog and Oct4 promoter was decreased compared to naïve rMSCs. The ratio of methylated CpG loci of both Oct4 and Nanog was significantly increased when rMSCs and De-Os-rMSCs were subjected to osteogenic induction (Supplementary Figure-2). It should be noted that, when we counted the percentage of methylation on CpG locus of Oct4 and Nanog independently, some CpG locus were specifically demethylated in De-Os-MSCs (Fig. 5E,F). Taken together, these data suggest that DNA demethylation could be involved, at least partially, in the regulation of pluripotent gene expression and de-differentiation of MSCs.

Histone modification is principal epigenetic machinery linked to the establishment and maintenance of transcriptional states of genes^33^,^34^. Histone-modifying enzymes are involved in the addition or removal of histone modifications, and reciprocally collaborate to compile the complex “histone code” to fine-tune epigenetic context at a specific regulatory region, modulating the gene expression^35^. To investigate the possible involvement of histone modifications in the regulation of pluripotent genes, a focused qRT-PCR array encompassing 84 key genes encoding enzymes known to modify genomic DNA and histones was used. As shown in Fig. 5G, 16/84 histone or DNA modifying enzymes (19%) were differentially expressed between MSCs and De-Os-MSCs. The expression profiles of some of these genes were confirmed by RT-PCR analysis (Fig. 5H). It is likely that complex histone modification mechanisms may contribute to the difference between MSCs and De-Os-MSCs. Since KDM5C, which is a specific histone 3 lysine 4 demethylase, is the most significantly downregulated gene, we decided to explore its involvement in maintaining histone 3 methylation status and regulation of pluripotent genes in De-Os-MSCs. Our western blot analysis showed that the global expression level of H3K4me2 and H3K4me3 was dramatically increased in De-MSCs (Fig. 5I), probably due to the decreased expression of KDM5C. Since H3K4me3 occupancy on promoter region is associated with target gene activation in stem cells, we next performed chromatin immunoprecipitation (CHIP) assay to check the occupancy of H3K4me3 on the promoter regions of Oct4 and Nanog with specific monoclonal antibody. Our results showed that the dedifferentiation process significantly increased the H3K4me3 occupancy on Oct4 and Nanog promoters (Fig. 5J–L). The increased occupancy of H3K4me3 is specific, since we did not find any significant changes in H3K27me3 binding on these promoters. Given the functional impact of acetylated histones is reduction to the nucleosomal barrier, binding of promoting cofactors, and thereby increasing transcriptional activity, we further determined histone acetylation mark on the promoter regions of Oct4 and Nanog in both MSCs and De-Os-MSCs by using anti-acetyl-H4 antibody (H4ac). Consistently, we found the occupancy of H4ac on the promoter regions of both Oct4 and Nanog was markedly increased in De-Os-MSCs compared to naïve MSCs (Fig. 5J–L). The induction of active but not suppressive histone marks on the promoter of pluripotent genes indicated gene-specific chromatin configuration was related to their increased expression.

### Nanog was indispensisbe for enhanced cell survival and osteogenic differentiation in De-Os-rMSCs

**Figure 6:**
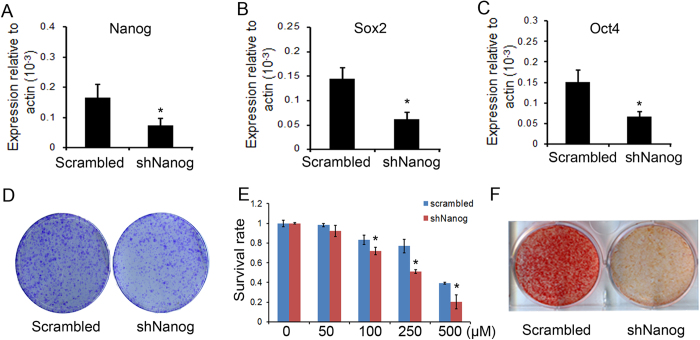
The effect of Nanog silencing on dedifferentiation-mediated reprograming. (**A**-**C**) Total RNA was extracted from rMSCs infected with shNanog or scrambled control. QRT-PCR was performed to evaluate the efficiency of Nanog silencing, and also the effect of Nanog silencing on gene expression levels of Oct4 and Sox2. β-actin was used as an internal control. The data are expressed as mean ± SD (n = 3). *p < 0.05. (**D**) Clongenic assay. The control and shNanog infected rMSCs were induced to undergo osteogenic differentiation and dedifferentiation for 10 days, and then plated at 1000 cells per 20 cm^2^ dish and cultured for 10 days, respectively. The cells were then stained with 0.5% crystal violet to view the colonies. (**E**) Nanog silencing influenced cell survival. The control and shNanog infected rMSCs were induced to undergo differentiation and dedifferentiation for 10 days and plated in 96-well plates. At days 3, these cells were challenged with 0–500 μM H_2_O_2_ for 12 h. Then, the cells were incubated with Alamar Blue for 2.5 hours at 37 °C. The metabolic rate of the cells was determined at 570 nm, with reference wavelength at 600 nm. Values are expressed as mean ± SD of three independent experiments. *p < 0.05. (**F**) The control and shNanog infected rMSCs were induced to undergo differentiation and dedifferentiation for 10 days, and plated in OIM for 10 days, the calcium deposits were stained with Alizarin Red S.

Our data so far have suggested the possible involvement of pluripotency-related genes in the dedifferentiation process. And we also found that De-Os-rMSCs could maintain the osteogenic differentiation advantage and the higher expression of pluripotency-related genes as well as osteogenesis-related genes for at least 14 days (Supplementary Figure 3). Given the fact that Nanog promoter harbors much lower DNA methylation than Oct4 in MSCs, and Nanog has been shown to be critical for maintaining MSCs potency and osteogenic differentiation in vivo^21^,^22^,^36^, we speculated that Nanog might be critical in the achievement of dedifferentiation phenotype. To test this, we constructed two lentiviral-shRNAs constructs targeting different sequences of Nanog in MSCs. QRT-PCR was conducted to evaluate the silencing efficiency of the two shRNAs, and the result showed that shNanog-1 reduced the mRNA level of Nanog to about 45% of the control (Fig. 6A), while shNanog-2 had no effect (data not shown). Interestingly, after shNanog knockdown, the expression level of Oct4 and Sox2 was significantly decreased (Fig. 6B,C). Most importantly, the enhanced colony forming ability, cell survival and osteogenic differentiation potential exhibited by De-Os-MSCs were completely reversed by Nanog silencing (Fig. 6D–F), indicating the advantageous phenotypes induced by dedifferentiation-mediated reprogramming are mostly, if not completely, attributed to integrated Nanog/Oct4-Sox2 regulatory network.

**Figure 7:**
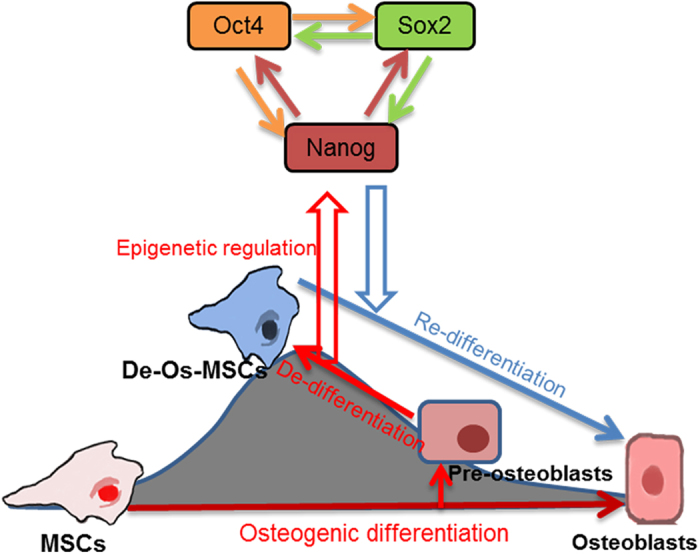
The schematic outlines of the procedure for de-osteogenic differentiation of MSCs and involvement of Nanog/Oct4 in this process.

Taken together, our data demonstrated that De-Os-rMSCs showed advantages over untreated rMSCs when priming with osteogenic induction medium, in terms of clonogenicity, proliferation, cell survival, migration and osteogenic differentiation. And epigenetic regulation of Oct4 and Nanog leaded to the increases of Oct4 and Nanog in De-Os-rMSCs, which may partially contribute to the advantages exhibited by De-Os-rMSCs. The epigenetic regulation and involvement of Nanog/Oct4 in the de-osteogenic differentiation of MSCs are schematically illustrated in Fig. 7.

## Discussion

In the present study, we showed that after early commitment into osteogenic differentiation, re-incubation in the normal media could revert the committed cells back to a stem cell-like state. These dedifferentiated MSCs resembled original MSCs by their morphology, characteristic cell surface markers and multipotent differentiation capacity. However, as seen before with neurogenic differentiation, De-Os-MSCs reveal distinguishing stem cell phenotype, such as enhanced osteogenic differentiation capacity, cell survival and colony forming ability, as compared with naïve MSCs. More significantly, De-Os-MSCs-inoculated nude mice harbored more bone-like tissue after cell transplantation, indicating increased propensity to bone formation in vivo. These results indicated that dedifferentiation could be achieved after different lineage commitment and reinforced the potential therapeutic benefit of in vitro dedifferentiation strategy, which could have broad impact on the application of MSCs in regenerative medicine.

Transcription factors, such as Oct4, Nanog and Sox2, are essential for the maintenance of ESC pluripotency and reprogramming of somatic cells into iPS cells^31^. These factors are also expressed in BM-MSCs, suggesting a similar regulatory role although their expression levels are significantly lower than that in ESCs. It has been reported that these transcription factors target similar genes in MSCs and ESCs, and regulate MSCs cell cycle progression, plasticity and self-renewal^21^,^22^,^32^,^37^. In the current study, we demonstrated that the expression levels of Oct4, Nanog and Sox2 were significantly upregulated after dedifferentiation. Knockdown of Nanog dramatically reversed the enhanced cell survival, colony formation and osteogenic differentiation observed in De-Os-MSCs, indicating dedifferentiation-induced enhancement of MSCs potency was attributed to increased expression of Nanog. It has been shown previously that Nanog is expressed during surgically induced bone marrow formation and is functionally involved in post natal marrow stromal cell maintenance and differentiation^36^. Intriguingly, very recent study in adult endothelial cells (EC) illustrated that Nanog played essential role in the dedifferentiation of EC toward adult stem cells^38^. These observations, together with our findings showing dedifferentiation-induced expression of Nanog and reversion of dedifferentiation phenotype by Nanog silencing, suggest that Nanog is the key transcription factor controlling MSCs identity and fate conversion.

While the functions of pluripotent transcription factors have been evaluated in MSCs^21^,^22^,^32^,^37^, few studies have focused on the roles of epigenetic regulation in controlling these gene expression and MSCs identity. DNA methylation is the most frequent epigenetic mechanism controlling gene expression. On the other hand, it was recently proposed that the dedifferentiation of Müller cells that preceded the regenerative response to injury in zebrafish was driven by changes in DNA methylation^39^. In this study, using bisulfite sequencing analysis, we observed that the Oct4 promoter was hypermethylated (>70%) whereas Nanog promoter hypomethylated (13%) in untreated MSCs. It is well-known that ESCs are predominantly demethylated on Oct4 and Nanog promoters (5%). And the methylation at Oct4 and Nanog promoter in MSCs is greater than the ESCs^40^. Our result also showed that compared to ESCs, the degree of methylation at Oct4 loci in MSCs was much greater, whereas the degree of Nanog methylation was comparable, inferring that Oct4 expression in MSCs may be more restricted by DNA methylation than Nanog. More interestingly, though the general methylation of Oct4 promoter showed mild fluctuation after dedifferentiation, the dedifferentiation process significantly decreased the methylation of Nanog promoter compared to untreated rMSCs (6.7% Vs 13.3%), which is similar to ESCs. It is noteworthy that when we counted the percentage of methylated CpG at first CpG of Nanog independently, we found that methylation in this locus was more significantly decreased after dedifferentiation. Taken together, these results indicated that DNA methylation was involved in the regulation of Nanog expression during fate conversion of MSCs.

In our previous study, we observed the differences in histone methylation between MSCs and dedifferentiated MSCs from neuron-like cells^14^, suggesting that histone modification are probably involved in the dedifferentiation-mediated reprogramming of MSCs. In ES cells, genes that are involved in early lineage commitment maintain both repressive (H3K27me3) and activating (H3K4me3) histone modifications^25^. These bivalent genes are considered to be poised for rapid activation in response to appropriate differentiation signals^41^. For MSCs, while it is still unclear whether chromatin structure and histone-modifying enzymes utilize the similar mechanisms to modulate gene expression, emerging evidence indicates that histone modifications play an important role in the cell fate determination of MSCs^23^,^42^,^43^,^44^. It has been shown that acetylation modification patterns are changed in MSCs during in vitro culturing^45^. And the higher ratio of H3K4me3 to H3K27me3 at promoter of PPARγ2 is correlated with diminished promoter activity in late passage cells exposed to adipogenic stimuli^46^. In particular, histone demethylases, such as KDM6A, KDM4B and KDM6B have been discovered recently to play a critical role in MSC cell fate commitment by removing H3K9me3 and H3K27me3 on different sets of lineage-specific genes, providing the first demonstration that histone demethylation controls the lineage determination in MSCs^42^,^43^. Upregulation of KDM6B during osteogenesis has also been shown to be associated with adoption of osteogenic lineage^47^. Interestingly, in this study, we have identified that KDM5C, a newly identified member of the KDM5 family of specific H3K4 demethylases, is dramatically downregulated in De-Os-rMSCs compared to unmanipulated rMSCs. In keeping with this observation, we have also found that the global expression level of H3K4 methylation is significantly increased after dedifferentiation. It has been shown recently that KDM5C can be recruited to both enhancer and promoter elements in ESC and in neuronal progenitor cells. Knockdown of KDM5C deregulates transcription via local increases in H3K4me3^48^. To further elucidate the role of histone modification in specific gene regulation, we examined histone H3 methylation in K4 and K27 on the promoter regions of Nanog and Oct4 in MSCs and De-Os-MSCs. Compared to rMSCs, De-Os-rMSCs showed significantly increased histone H3 methylation levels in K4, but not K27, implying the activation of gene transcription. Thus, our data provide the first documentation that KDM5C is associated with activating H3K4 expression level and regulation of MSC stemness. On top of that, we have observed that multiple histone acetyltransferases, such as HAT1, Esco1, Esco2, KAT6A, KAT6B, is markedly increased in De-Os-rMSCs by our focused PCR array. In corroboration with this result, we have found that compared to rMSCs, De-Os-rMSCs showed significantly increased histone H4 acetylation occupancy on the promoters of pluripotent genes. Based on these findings, while the final biological significance of these histone acetyltransferases in the complex dedifferentiation-mediated-reprogramming of MSCs is still elusive, we speculate that increased histone acetylation of key pluripotency genes might contribute to the gene activation and enhanced stemness in De-Os-rMSCs. Taken together, it appears that increased occupancy of activating H3K4 and H4ac leading to opening of the chromatin structure, make De-Os-MSCs poised for rapid activation of stemness genes.

In closing, the present study has revealed that epigenetic memory in De-Os-MSCs gained by priming with osteogenic induction medium favored their differentiation along osteoblastic lineage with improved cell survival and migratory abilities. Epigenetic mechanisms involving both DNA methylation and histone modification at promoters of Nanog and Oct4 endowed MSCs with epigenetic plasticity by opening of the chromatin structure when differentiated cells were primed to change cell fate and acquired multipotency. With easy culture manipulation and low tendency of tumor formation, dedifferentiation strategy provides a feasible approach to enhance therapeutic efficacy in stem cell based-regenerative medicine. Focused efforts on the detailed mechanisms linking the epigenetic regulatory mechanisms to adult stem cell fate conversion in physiological/pathological conditions may assist us to develop and recognize reagents that are able to efficiently promote this cellular dedifferentiation strategy which has the potential to enhance the regenerative capacity in mammals.

## Materials and Methods

### Culture of rat MSCs

All experiments were approved by the Animal Research Ethics Committee of the authors’ institution and carried out in accordance with the approved guidelines. Rat bone marrow MSCs (rMSCs) were isolated and expanded as previously described^49^. The rMSCs were cultured in α-MEM medium supplemented with 10% fetal bovine serum (FBS), 100 U/mL penicillin, 100 mg/mL streptomycin, and 2 mM L-glutamine (all from Invitrogen Corporation, Carlsbad, CA) and cultured at 37 °C, 5% CO_2_.

### Plasmid construction

Two different shRNAs were chosen from rat Nanog mRNA sequence, which target nucleotides 2254–2276 and 752–774 respectively, and one nonspecific shRNA was designed as control. The two Nanog shRNA target sequences are: 5’– GAG GCT TCT ATG TTA ATA T-3’ and 5’– GCT ATT CTC AGG GCT ATC T-3’. The synthesized oligos were annealed and ligated into the HpaI/XhoI sites of the pLL3.7 vector.

### Lentivirus production

Pseudo-lentivirus was produced by transient transfection of 293FT packaging cells (Invitrogen, USA) using the calcium phosphate method. Culture supernatants were harvested at 48 and 72 hours after transfection and lentiviral particles were concentrated using PEG6000^50^. For transduction, 1 × 10^5^ cells were seeded into 6-well plate and incubated with lentivirus and 8 μg/mL polybrene in the incubator for 24 h.

### Induction of osteogenic differentiation, de-differentiation, and re-differentiation of rMSCs

To initiate osteogenic differentiation, MSCs at p3-p8 were transferred to osteogenic induction media (OIM) containing basal media with 1 nM dexamethasone, 50 μM ascorbic acid, and 20 mM β-glycerolphosphate (all from Sigma-Aldrich) for 7–10 days (Os-MSCs). After osteogenic induction, osteogenic media was replaced with basal media and allowed to grow for another 7–10 days (De-Os-MSCs). Then the media were removed and the cells were washed with PBS, and transferred to osteogenic again for 7–10 days (Re-Os-MSCs) depending on the following assays.

### Phenotypic characterization of rMSCs and De-Os-rMSCs

After reaching 80% confluence, the rMSCs were rinsed twice with phosphate buffered saline (PBS) and treated with 0.05% trypsin-EDTA for 2 minutes. Then, serum-containing medium was immediately added to the culture to end trypsinization. Then, the fluid was collected and centrifuged (800 g for 5 minutes). After discarding the supernatant, the precipitate was resuspended in staining buffer and incubated with fluorochrome-conjugated primary antibodies against CD31, CD34, CD45, CD73, CD90 or corresponding isotype control (BD Biosciences, USA) at 4 °C for 30 minutes. The stained cells were immediately detected using Flow Cytometry (BD Biosciences, USA).

### Osteogenic differentiation

MSCs and De-Os-MSCs were plated at 4 × 10^3^ cells/cm^2^ in a 24-well plate and cultured in the basal medium until the cells reached confluence, respectively. They were then incubated in OIM, which is basal medium supplemented with 1 nM dexamethasone, 50 μM ascorbic acid, and 20 mM β-glycerolphosphate (all from Sigma-Aldrich) at 37 °C, 5% CO_2_ as described previously. At day 10, the mineralization of MSCs and De-Os-MSCs was assessed by Alizarin red S staining. Briefly, to evaluate the mineralized nodule formation in vitro, the cell/matrix layer was washed with the PBS, fixed with 70% ethanol for 10 min, and stained with 0.5% Alizarin red S (pH 4.1; Sigma, St. Louis, MO) for 5 min.

### Adipogenic differentiation

MSCs were plated at 4 × 10^3^ cells/cm^2^ in a 6-well culture plate and cultured until the cells reached confluence. The medium was then replaced with adipogenic medium, which is basal medium supplemented with 500 nM dexamethasone, 0.5 mM isobutylmethylxanthine, 50 mM indomethacin, and 10 mg/mL of insulin (all from Sigma-Aldrich). The cells were cultured for another 21 days, then the cells were fixed with 70% ethanol for 10 minutes, stained with 0.3% fresh Oil Red O solution (Sigma-Aldrich) for 10 mins. The wells were rinsed 3 times with distilled water and viewed using a LEICA Q500MC microscope (Leica Cambridge Ltd).

### Chondrogenic differentiation

For chondrogenic differentiation, a micromass culture system was used. 5 ul MSCs at 1.6 × 10^7^ cells/mL were dropped in the central of a 24-well plate, respectively. The plates were placed in incubator at 37 °C, 5% CO_2_ without culture medium for 2 hours. Then, these cells were cultured in chondrogenic medium, which is basal medium supplemented with 10 ng/mL transforming growth factor-β3 (R&D Systems), 500 ng/mL bone morphogenetic protein-2 (R&D Systems), 10^−7^ M dexamethasone, 50 mg/mL ascorbate-2-phosphate, 40 mg/mL proline, 100 mg/mL pyruvate (all from Sigma-Aldrich), and 1:100 diluted ITS + Premix (6.25 mg/mL insulin, 6.25 mg/mL transferrin, 6.25 mg/mL selenous acid, 1.25 mg/mL bovine serum albumin, and 5.35 mg/mL linoleic acid) (Becton Dickinson, Franklin Lakes, NJ). Chondrogenic medium was changed every 3 days. At day 14, the deposition of glycoaminoglycans (GAG) was assessed by alcian blue staining. The cell/matrix layer was washed with PBS, fixed with 70% ethanol, stained with 3% acetic acid (pH 2.5) for 3 minutes and then incubated with 1% alcian blue solution (alcian blue 8GX 1 g / 3% acetic acid 100 ml, pH 2.5; Sigma Aldrich, St Louis, MO, USA) for 30 minutes at room temperature. After washing with distilled water, the positive stain was viewed under a phase-contrast microscope (Leica Microsystems Wetzlar GmbH, Wetzlar, Germany).

### Colony forming ability (CFA) assay

The cells were plated at 100–2000 cells per 20 cm^2^ dish and cultured for 10 days. The cells were stained with 0.5% crystal violet (Sigma, St. Louis, MO) to count the number of cell colonies. Colonies that were smaller than 2 mm in diameter and faintly stained were ignored.

### Cell proliferation assay

The cells were plated at 2,000 cells/well in the complete culture medium in a 96-well plate and incubated at 37 °C, 5% CO_2_. At day 3, cell proliferation was assessed using the BrdU assay kit (Roche Applied Science, Indianapolis, IN) according to the manufacturer’s instruction. Briefly, the cells were labeled with 10 μL BrdU for 3 h at 37 °C. The labeling medium was then removed and then fixed with 100 μL FixDenat solution. After removing FixDenat, 100 μL peroxidase-conjugated anti-BrdU antibodies were added to each well and incubated with the cells for 90 min at room temperature. After washing with PBS, 100 μL of a substrate solution [3, 3, 5, 5–tetramethylbenzidine dissolved in 15% (v/v) dimethylsulfoxide] was added to each sample for 30 min at room temperature. The absorbance at 450 nm was measured and reported.

### Alamar Blue assay

The cell survival of rMSCs and De-Os-rMSCs was determined using alamarBlue cell viability reagent (Invitrogen). The rMSCs and De-Os-rMSCs were seeded at 5,000 cells/well in a 96-well plate and incubated at 37 °C, 5% CO_2_, respectively. At day 3, these cells were challenged with 0–500 μM H_2_O_2_ for 12 or 24 hours, respectively. Then, the cells were incubated with alamarBlue for 2.5 hours at 37 °C. The metabolic rate of the cells was determined at 570 nm, with reference wavelength at 600 nm.

### Transwell migration assay

MSCs and De-Os-MSCs (2 × 10^4^ cells/well) were inoculated into the upper layer of a transwell insert in α-MEM with 2% FBS. 20% FBS containing α-MEM was placed at the bottom layer (BD Falcon, Cat. 353503). After incubating for 12 hours at 37 °C, 5% CO_2_, MSCs at the upper layer of membrane were scraped and MSCs at the lower layer were stained with 0.05% crystal violet (Sigma, St. Louis, MO) and photographed under an inverse phase contrast microscope. The number of cells was quantified in the randomly selected fields.

### Ectopic bone formation

In vivo studies were performed with the approval of the Animal Experimentation Ethics Committee of The Chinese University of Hong Kong. After anesthesia, an incision was made on the dorsum and a subcutaneous pocket was created. 2.5 × 10^6^ MSCs or De-Os-MSCs were seeded onto sterilized Skelite® resorbable Si-TCP bone graft substitute, and Si/TCP cubes with PBS was served as control group. Then they were transplanted into the same mice. The wound was then closed in layers. At week 8, the scaffold with and without cells were harvested for H&E staining, as well as immohistochemical staining of collagen type I and osteocalcin (OCN).

### Quantitative real time RT-PCR (qRT-PCR)

The cells were harvested and homogenized for RNA extraction with RNeasy mini kit (Qiagen, Hilden, Germany). The mRNA was reverse-transcribed to cDNA by the PrimeScript First Strand cDNA Synthesis Kit (Takara). 5 μl of total cDNA of each sample were amplified in a final volume of 25 μl of reaction mixture containing Platinum SYBR Green, qPCR SuperMix-UDG ready-to-use reaction cocktail and specific primers using the ABI StepOne Plus system (all from Applied Biosystems, CA, USA). The expression of target gene was normalized to that of β-actin gene which was shown to be stable in this study. Relative gene expression was calculated with the 2^−△CT^ formula. The sequences of the primers were shown in Supplementary Table 1.

### DNA isolation and bisulfite treatment

Genomic DNA was isolated from MSCs using PureLink^®^ Genomic DNA isolation kit following the manufacturer’s instructions (Invitrogen). Bisulfite modification was done as described previously^51^. Briefly, about 2 μg of genomic DNA was denatured by NaOH (final concentration, 0.2 mol/L) for 10 min at 37 °C. Hydroquinone and sodium hydroxide were added, and samples were incubated at 50 °C for 16 h. Modified DNA was purified using Wizard DNA Clean-Up System following the manufacturer’s instructions (Promega) and eluted into 50 μL water. DNA was treated with NaOH (final concentration, 0.3 mol/L) for 5 min at room temperature, ethanol precipitated, and resuspended in 20 μL water. Modified DNA was used immediately or stored at −20 °C.

### Bisulfite sequencing

Bisulfite-modified genomic DNA was amplified by PCR. All PCRs were done using KAPA2G™ Fast HotStart DNA Polymerase Polymerase. The sequences of primers used for the bisulfite sequencing analysis were shown in Supplementary Table 2. PCR products were run on 1.5% agarose gels and bands were excised using TaKaRa MiniBEST Agarose Gel DNA Extraction Kit following the manufacturer’s instructions (TaKaRa). Purified bands were cloned using pMD™19-T Vector Cloning Kit following the manufacturer’s instructions (TaKaRa). Colonies were selected and grown overnight in Luria-Bertani medium containing ampicillin (100 μg/mL) with shaking at 37 °C. Plasmid DNA was isolated using TaKaRa MiniBEST Agarose Gel DNA Extraction Kit following the manufacturer’s instructions (TaKaRa). Plasmids were sequenced using the M13 universal reverse primer (BGI).

### PCR Array

Total RNA was isolated using the RNeasy mini kit according to manufacturer’s instructions (Life Technologies, Carlsbard, CA). The quantity of RNA was determined by spectrophotometry at 260 nm and the OD 260/280 ratio was measured to determine the quality. One microgram total RNA of each sample was then transcribed into cDNA using RT^2^ First Strand Kit (Qiagen, Hilden, Germany). Real-time PCR array analysis was performed according to manufacturer’s protocol with the RT^2^ Real-Time SYBR green PCR Master Mix on Applied Biosystems 7500 Fast Real-Time PCR System. Expression of 84 genes was analyzed using the histone modifying enzyme array (MeAH-511A). Data were normalized using multiple housekeeping genes and analyzed by comparing 2^−Δ*Ct*^ of the normalized data. Fold changes were calculated relative to the untreated MSCs.

### Western blot

Equal proteins were loaded onto 10% Tris/glycine gels for electrophoresis and then transferred to a PVDF membrane (Millipore). Anti-H3K4me2 (Millipore, 1:1000), anti-H3K4me3 (Millipore, 1:1000), anti-β-catenin (BD, 1:1000), anti-ERK1/2 (BD, 1:2000), anti-p-ERK1/2 (BD, 1:1000), anti-JNK/SAPK (BD, 1:1000) or anti-GAPDH (Santa Cruz, 1:1000) antibodies were used in this study. After washing in TBST, the membrane was incubated with horseradish peroxidase-linked secondary antibodies (anti-mouse or anti-goat) for 1 h at room temperature. Following TBST washes, protein was detected with the enhanced chemiluminescence blotting reagents (Amersham Biosciences) according to the manufacturer’s instruction.

### Chromatin immunoprecipitation

CHIP assay was conducted according to Millipore Magna CHIP kit (Catalogue Number: 17–10085). Briefly, all of the subsequent steps were performed at 0–4 °C. MSCs (2 × 10^7^) were washed with phosphate-buffered saline (pH 7.4) and lysed for 15 min with rotation 800 g at 4 °C for 5 minutes. Cell pellets were collected and resuspended in 0.5 mL of Nuclear Lysis Buffer. Then samples were sonicated on ice using ULTRASONIC PROCESSOR (Model: GE 130PB). Afterward, IP was performed with 2 μg antibody and 2 μg rabbit IgG as negative control. Washes and purification of the CHIP DNA were performed as suggested by manufacturer. Real-time PCR were performed using 1 μl of CHIP DNA solution, and target DNA levels in the IP were normalized to respective target levels in the input DNA. Antibody used for CHIP: Anti-acetyl-Histone H4 Antibody (Millipore, 06–866); Trimethyl-Histone H3 (Lys4) (contain rabbit IgG) (Millipore, 17–614); Trimethyl-Histone H3 (Lys27) (Millipore, 17–622). ChIP PCR analysis primer sets shown in Supplementary Table 3.

### Histology and immunohistochemistry

Immunohistochemical staining was performed as previously described^52^. The scaffold without and with cells were washed in PBS, fixed in 4% paraformaldehyde, decalcified dehydrated and embedded in paraffin. Sections were cut at a thickness of 5μm and were stained with H&E after deparaffination. Endogenous peroxidase activity was quenched with 3% hydrogen peroxide for 20 minutes at room temperature. Antigen retrieval was then performed with citrate buffer at 80 °C for 10 minutes for collagen type I and osteocalcin detection. Primary antibodies against collagen type I (1:100; sc-8784; Santa Cruz, CA, USA) and osteocalcin (1:100; sc-365797, Santa Cruz, CA, USA) were used. Donkey anti-goat IgG horseradish peroxidase (HRP)-conjugated secondary antibody and goat anti mouse horseradish peroxidase (HRP)-conjugated secondary antibody (sc-2020, sc-2302, Santa Cruz Biotechnology, CA, USA; both at a dilution of 1:100) was then added for an hour, followed by 3,3’ diaminobenzidine tetrahydrochloride (DAKO, Glostrup, Denmark) in the presence of H_2_O_2_ for signal detection of collagen type I and osteocalcin. Afterward, the sections were rinsed, counterstained in hematoxylin, dehydrated with graded ethanol and xylene, and mounted with p-xylene-bis-pyridinium bromide (DPX) permount (Sigma Aldrich, St Louis, MO, USA). Primary antibody was replaced with blocking solution in the negative controls. All incubation times and conditions were strictly controlled. The sections were examined under light microscopy (DMRXA2, Leica Microsystems Wetzlar GmbH, Germany).

### Data analysis

Data were presented as mean ± SD and shown in boxplots. Comparison of two independent groups was done using Mann-Whitney U test while comparison of more than two independent groups was done using Kruskal-Wallis test followed by post-hoc pairwise comparison. All the data analysis was done using SPSS (version 16.0; SPSS Inc, Chicago, IL). p < 0.05 was regarded as statistically significant.

## Additional Information

**How to cite this article**: Rui, Y. *et al.* Epigenetic memory gained by priming with osteogenic induction medium improves osteogenesis and other properties of mesenchymal stem cells. *Sci. Rep.* **5**, 11056; doi: 10.1038/srep11056 (2015).

## Supplementary Material

Supplementary Information

## References

[b1] Dimitriou R., Jones E., McGonagle D. & Giannoudis P. V. Bone regeneration: current concepts and future directions. BMC Med 9, 66 (2011). 21627784 10.1186/1741-7015-9-66 PMC3123714

[b2] Kon E. *et al.* Bone regeneration with mesenchymal stem cells. Clin Cases Miner Bone Metab 9, 24–27 (2012). 22783331 PMC3392668

[b3] Parekkadan B. & Milwid J. M. Mesenchymal stem cells as therapeutics. Annu Rev Biomed Eng 12, 87–117 (2010). 20415588 10.1146/annurev-bioeng-070909-105309 PMC3759519

[b4] Hicks A. U. *et al.* Transplantation of human embryonic stem cell-derived neural precursor cells and enriched environment after cortical stroke in rats: cell survival and functional recovery. Eur J Neurosci 29, 562–574 (2009). 19175403 10.1111/j.1460-9568.2008.06599.x

[b5] Li Y. *et al.* Human marrow stromal cell therapy for stroke in rat: neurotrophins and functional recovery. Neurology 59, 514–523 (2002). 12196642 10.1212/wnl.59.4.514

[b6] Swanger S. A., Neuhuber B., Himes B. T., Bakshi A. & Fischer I. Analysis of allogeneic and syngeneic bone marrow stromal cell graft survival in the spinal cord. Cell Transplant 14, 775–786 (2005). 16454352 10.3727/000000005783982594

[b7] Chen J. *et al.* Intravenous administration of human umbilical cord blood reduces behavioral deficits after stroke in rats. Stroke 32, 2682–2688 (2001). 11692034 10.1161/hs1101.098367

[b8] Jiang Y. *et al.* Pluripotency of mesenchymal stem cells derived from adult marrow. Nature 418, 41–49 (2002). 12077603 10.1038/nature00870

[b9] Joshi C. V. & Enver T. Plasticity revisited. Curr Opin Cell Biol 14, 749–755 (2002). 12473350 10.1016/s0955-0674(02)00392-7

[b10] Petersen B. E. *et al.* Bone marrow as a potential source of hepatic oval cells. Science 284, 1168–1170 (1999). 10325227 10.1126/science.284.5417.1168

[b11] Woodbury D., Schwarz E. J., Prockop D. J. & Black I. B. Adult rat and human bone marrow stromal cells differentiate into neurons. J Neurosci Res 61, 364–370 (2000). 10931522 10.1002/1097-4547(20000815)61:4<364::AID-JNR2>3.0.CO;2-C

[b12] Liu Y. *et al.* Switching from bone marrow-derived neurons to epithelial cells through dedifferentiation and translineage redifferentiation. Cell Biol Int 34, 1075–1083 (2010). 20939829 10.1042/CBI20100516

[b13] Poloni A. *et al.* Human dedifferentiated adipocytes show similar properties to bone marrow-derived mesenchymal stem cells. Stem Cells 30, 965–974 (2012). 22367678 10.1002/stem.1067

[b14] Liu Y. *et al.* Dedifferentiation-reprogrammed mesenchymal stem cells with improved therapeutic potential. Stem cells 29, 2077–2089 (2011). 22052697 10.1002/stem.764

[b15] Uccelli A., Moretta L. & Pistoia V. Mesenchymal stem cells in health and disease. Nat Rev Immunol 8, 726–736 (2008). 19172693 10.1038/nri2395

[b16] Blondheim N. R. *et al.* Human mesenchymal stem cells express neural genes, suggesting a neural predisposition. Stem Cells Dev 15, 141–164 (2006). 16646662 10.1089/scd.2006.15.141

[b17] Barzilay R., Melamed E. & Offen D. Introducing transcription factors to multipotent mesenchymal stem cells: making transdifferentiation possible. Stem Cells 27, 2509–2515 (2009). 19591229 10.1002/stem.172

[b18] Spivakov M. & Fisher A. G. Epigenetic signatures of stem-cell identity. Nat Rev Genet 8, 263–271 (2007). 17363975 10.1038/nrg2046

[b19] Papp B. & Plath K. Epigenetics of reprogramming to induced pluripotency. Cell 152, 1324–1343 (2013). 23498940 10.1016/j.cell.2013.02.043 PMC3602907

[b20] Marks H. & Stunnenberg H. G. Transcription regulation and chromatin structure in the pluripotent ground state. Biochim Biophys Acta 1839, 129–137 (2014). 24096207 10.1016/j.bbagrm.2013.09.005

[b21] Liu T. M. *et al.* Effects of ectopic Nanog and Oct4 overexpression on mesenchymal stem cells. Stem Cells Dev 18, 1013–1022 (2009). 19102659 10.1089/scd.2008.0335 PMC3190294

[b22] Go M. J., Takenaka C. & Ohgushi H. Forced expression of Sox2 or Nanog in human bone marrow derived mesenchymal stem cells maintains their expansion and differentiation capabilities. Exp Cell Res 314, 1147–1154 (2008). 18187129 10.1016/j.yexcr.2007.11.021

[b23] Ye L. *et al.* Histone demethylases KDM4B and KDM6B promotes osteogenic differentiation of human MSCs. Cell Stem Cell 11, 50–61 (2012). 22770241 10.1016/j.stem.2012.04.009 PMC3392612

[b24] Boquest A. C., Noer A. & Collas P. Epigenetic programming of mesenchymal stem cells from human adipose tissue. Stem Cell Rev 2, 319–329 (2006). 17848719 10.1007/BF02698059

[b25] Bernstein B. E. *et al.* A bivalent chromatin structure marks key developmental genes in embryonic stem cells. Cell 125, 315–326 (2006). 16630819 10.1016/j.cell.2006.02.041

[b26] Mikkelsen T. S. *et al.* Genome-wide maps of chromatin state in pluripotent and lineage-committed cells. Nature 448, 553–560 (2007). 17603471 10.1038/nature06008 PMC2921165

[b27] Zhao X. D. *et al.* Whole-genome mapping of histone H3 Lys4 and 27 trimethylations reveals distinct genomic compartments in human embryonic stem cells. Cell Stem Cell 1, 286–298 (2007). 18371363 10.1016/j.stem.2007.08.004

[b28] Pan G. *et al.* Whole-genome analysis of histone H3 lysine 4 and lysine 27 methylation in human embryonic stem cells. Cell Stem Cell 1, 299–312 (2007). 18371364 10.1016/j.stem.2007.08.003

[b29] Leu Y. W., Huang T. H. & Hsiao S. H. Epigenetic reprogramming of mesenchymal stem cells. Adv Exp Med Biol 754, 195–211 (2013). 22956503 10.1007/978-1-4419-9967-2_10

[b30] Hsiao S. H. *et al.* DNA methylation of the Trip10 promoter accelerates mesenchymal stem cell lineage determination. Biochem Biophys Res Commun 400, 305–312 (2010). 20727853 10.1016/j.bbrc.2010.08.048

[b31] Boyer L. A. *et al.* Core transcriptional regulatory circuitry in human embryonic stem cells. Cell 122, 947–956 (2005). 16153702 10.1016/j.cell.2005.08.020 PMC3006442

[b32] Tsai C. C., Su P. F., Huang Y. F., Yew T. L. & Hung S. C. Oct4 and Nanog directly regulate Dnmt1 to maintain self-renewal and undifferentiated state in mesenchymal stem cells. Mol Cell 47, 169–182 (2012). 22795133 10.1016/j.molcel.2012.06.020

[b33] Greer E. L. *et al.* Members of the H3K4 trimethylation complex regulate lifespan in a germline-dependent manner in C. elegans. Nature 466, 383–387 (2010). 20555324 10.1038/nature09195 PMC3075006

[b34] Bonasio R., Tu S. & Reinberg D. Molecular signals of epigenetic states. Science 330, 612–616 (2010). 21030644 10.1126/science.1191078 PMC3772643

[b35] Bannister A. J. & Kouzarides T. Regulation of chromatin by histone modifications. Cell Res 21, 381–395 (2011). 21321607 10.1038/cr.2011.22 PMC3193420

[b36] Bais M. V. *et al.* Role of Nanog in the maintenance of marrow stromal stem cells during post natal bone regeneration. Biochem Biophys Res Commun 417, 211–216 (2012). 22142851 10.1016/j.bbrc.2011.11.087 PMC3264057

[b37] Greco S. J., Liu K. & Rameshwar P. Functional similarities among genes regulated by OCT4 in human mesenchymal and embryonic stem cells. Stem Cells 25, 3143–3154 (2007). 17761754 10.1634/stemcells.2007-0351

[b38] Kohler E. E. *et al.* Low-Dose 6-Bromoindirubin-3’-oxime Induces Partial Dedifferentiation of Endothelial Cells to Promote Increased Neovascularization. Stem Cells 32, 1538–1552 (2014). 24496925 10.1002/stem.1658 PMC4037358

[b39] Reyes-Aguirre L. I. *et al.* Glutamate-induced epigenetic and morphological changes allow rat Muller cell dedifferentiation but not further acquisition of a photoreceptor phenotype. Neuroscience 254, 347–360 (2013). 24096137 10.1016/j.neuroscience.2013.09.048

[b40] Yannarelli G. *et al.* Brief report: The potential role of epigenetics on multipotent cell differentiation capacity of mesenchymal stromal cells. Stem Cells 31, 215–220 (2013). 23097343 10.1002/stem.1262

[b41] Guenther M. G. *et al.* Chromatin structure and gene expression programs of human embryonic and induced pluripotent stem cells. Cell Stem Cell 7, 249–257 (2010). 20682450 10.1016/j.stem.2010.06.015 PMC3010384

[b42] Hemming S. *et al.* EZH2 and KDM6A act as an epigenetic switch to regulate mesenchymal stem cell lineage specification. Stem Cells 32, 802–815 (2014). 24123378 10.1002/stem.1573

[b43] Musri M. M. *et al.* Histone demethylase LSD1 regulates adipogenesis. J Biol Chem 285, 30034–30041 (2010). 20656681 10.1074/jbc.M110.151209 PMC2943311

[b44] Dong R., Yao R., Du J., Wang S. & Fan Z. Depletion of histone demethylase KDM2A enhanced the adipogenic and chondrogenic differentiation potentials of stem cells from apical papilla. Exp Cell Res 319, 2874–2882 (2013). 23872478 10.1016/j.yexcr.2013.07.008

[b45] Zhu Y. *et al.* Alteration of Histone Acetylation Pattern during Long-Term Serum-Free Culture Conditions of Human Fetal Placental Mesenchymal Stem Cells. PLoS One 10, e0117068 (2015). 25671548 10.1371/journal.pone.0117068 PMC4324636

[b46] Lynch P.J. T. E., McGinnis K., Rovira Gonzalez Y. I., Lo Surdo J., Bauer S. R. & Hursh D. A. Chromatin changes at the PPAR-γ2 promoter during bone marrow-derived multipotent stromal cell culture correlate with loss of gene activation potential. Stem Cells **[Epub ahead of print]** (2015). 10.1002/stem.1967 25640287

[b47] Diaz P., Cuevas F. & Peralta O. A. GFP labelling and epigenetic enzyme expression of bone marrow-derived mesenchymal stem cells from bovine foetuses. Res Vet Sci 99, 120–128 (2015). 25637269 10.1016/j.rvsc.2014.12.019

[b48] Outchkourov N. S. *et al.* Balancing of histone H3K4 methylation states by the Kdm5c/SMCX histone demethylase modulates promoter and enhancer function. Cell Rep 3, 1071–1079 (2013). 23545502 10.1016/j.celrep.2013.02.030

[b49] Xu L. L. *et al.* Cellular retinol-binding protein 1 (CRBP-1) regulates osteogenenesis and adipogenesis of mesenchymal stem cells through inhibiting RXR alpha-induced beta-catenin degradation. Int J Biochem Cell B 44, 612–619 (2012). 10.1016/j.biocel.2011.12.018 22230368

[b50] Kutner R. H., Zhang X. Y. & Reiser J. Production, concentration and titration of pseudotyped HIV-1-based lentiviral vectors. Nat Protoc 4, 495–505 (2009). 19300443 10.1038/nprot.2009.22

[b51] Zinn R. L., Pruitt K., Eguchi S., Baylin S. B. & Herman J. G. hTERT is expressed in cancer cell lines despite promoter DNA methylation by preservation of unmethylated DNA and active chromatin around the transcription start site. Cancer Res 67, 194–201 (2007). 17210699 10.1158/0008-5472.CAN-06-3396

[b52] Rui Y. F., Lui P. P., Lee Y. W. & Chan K. M. Higher BMP receptor expression and BMP-2-induced osteogenic differentiation in tendon-derived stem cells compared with bone-marrow-derived mesenchymal stem cells. Int Orthop 36, 1099–1107 (2012). 22134708 10.1007/s00264-011-1417-1 PMC3337107

