# Supplementary material for: Epigenetic memory gained by priming with osteogenic induction medium improves osteogenesis and other properties of mesenchymal stem cells
Source: Sci Rep. 2015 Jun 8;5:11056. doi: 10.1038/srep11056 (PMC4459169; doi:10.1038/srep11056)
Supplement: Supplementary Information [file srep11056-s1.pdf]

Epigenetic memory gained by priming with osteogenic induction medium improves osteogenesis and other properties of mesenchymal stem cells

Yunfeng Rui[1, 2]*, Liangliang Xu[1]*, Rui Chen[3], Ting Zhang[1], Sien Lin[1], Yonghui Hou[1], Yang Liu[1], Fanbiao Meng[1], Zhenqing Liu[3], Ming Ni[1,9], Kam Sze Tsang[4], Fuyuan Yang[3], Chen Wang[2], Hsiao Chang Chan[3,5,7, 8], Xiaohua Jiang[3,5,7,8+], Gang Li [1, 5, 6,7,8+]

1. Department of Orthopaedics & Traumatology, The Chinese University of Hong Kong, Prince of Wales Hospital, Hong Kong SAR, PR China.
2. Department of Orthopaedics, Zhongda Hospital, Southeast University, 87 Ding Jia Qiao, Nanjing 210009, Jiangsu, PR China
3. Epithelial Cell Biology Research Center, School of Biomedical Sciences, Faculty of Medicine, The Chinese University of Hong Kong, Hong Kong SAR, PR China.
4. Department of Anatomical and Cellular Pathology, The Chinese University of Hong Kong, Hong Kong SAR, PR China.
5. Lui Che Woo Institute of Innovative Medicine, Faculty of Medicine, The Chinese University of Hong Kong, Hong Kong SAR, China.
6. Stem Cells and Regenerative Medicine Laboratory, Li Ka Shing Institute of Health Sciences, The Chinese University of Hong Kong, Hong Kong SAR, China.
7. Key Laboratory for Regenerative Medicine, Ministry of Education, School of Biomedical Sciences, The Chinese University of Hong Kong, Hong Kong SAR, PR China.
8. The Chinese University of Hong Kong, Shenzhen Research Institute, Shenzhen, PR China.
9. The Department of Orthopaedics, The General Hospital of Chinese People’s Liberation

Army, Beijing, PR China.

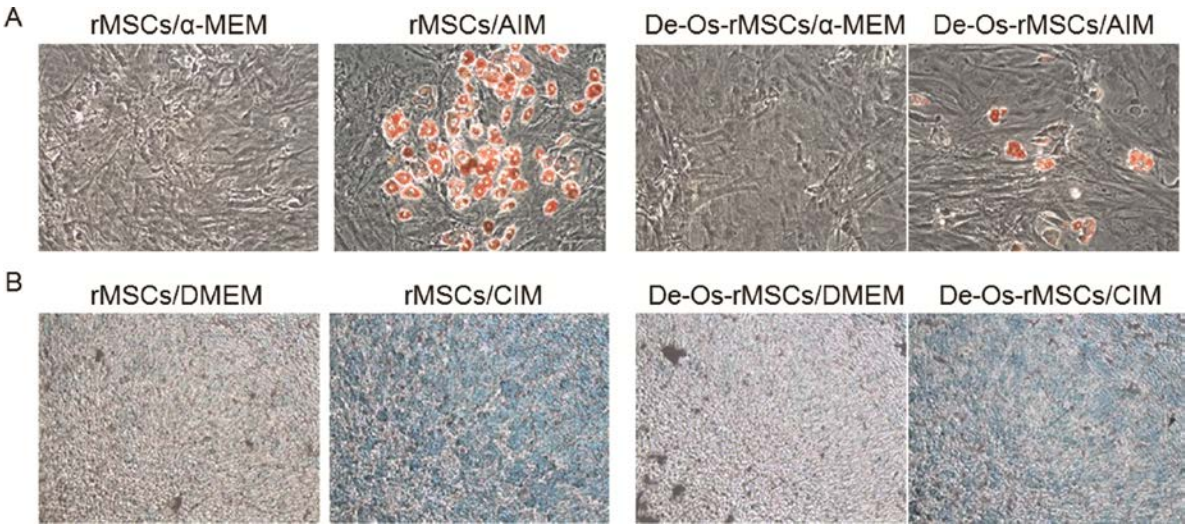

Supplementary Figure 1: De-Os-rMSCs showed lower adipogenic and chondrogenic differentiation potential. (A) Adipogenic differentiation assay. The untreated rMSCs and De-Os-rMSCs were plated in complete culture medium in a 6-well plate and incubated at 37°C with 5% CO_2, respectively. When the cells became confluent, the medium was changed with adipogenic induction medium. 21 days later, the cells were fixed and stained with Oil Red O. (B) Chondrogenic differentiation assay. The untreated rMSCs and De-Os-rMSCs were plated in a 24-well plate to form micromasses. Then the medium was changed with chondrogenic induction medium. 14 days later, the cells were fixed and stained with Alcian Blue.

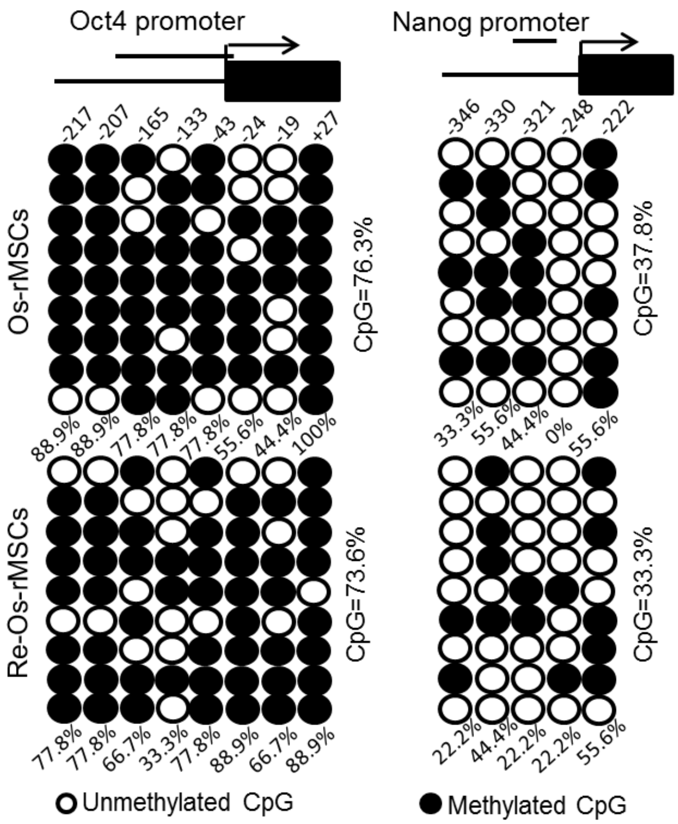

Supplementary Figure 2: DNA methylation status of Oct4 and Nanog promoters in Os-rMSCs and Re-Os-rMSCs using sodium bisulfite sequencing. The top panel indicates the CpG dinucleotide position of the Oct4 and Nanog promoter regions and the numbers show positions of CpGs relative to the translation start site. Each PCR product was subcloned and subjected to nucleotide sequencing analysis. Nine representative sequenced clones were depicted by filled (methylated) and open (unmethylated) circles for each CpG site.

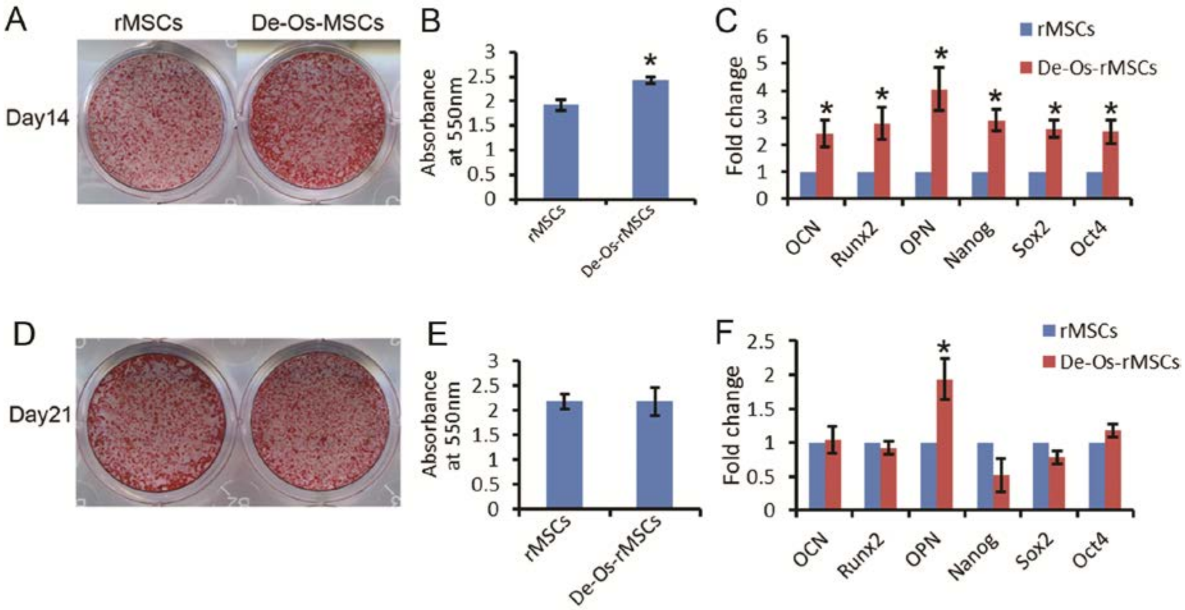

Supplementary Figure 3: De-Os-rMSCs maintained osteogenic differentiation advantage for at least 14 days. (A-C) De-Os-rMSCs were cultured in α-MEM for 14 days, then the cells were induced to differentiate in OIM for 10 days and stained with Alizarin Red S. The mineralization was quantified by extraction of Alizarin Red S dye with 10% CPC (B). *$p<0.05$. (C) QRT-PCR showing the expression of osteogenic differentiation genes and pluripotency-related genes.*$p<0.05$. (D-F) De-Os-rMSCs were cultured in α-MEM for 21 days, then the cells were induced to differentiate in OIM for 10 days and stained with Alizarin Red S. The mineralization was quantified by extraction of Alizarin Red S dye with 10% CPC (E). (F) QRT-PCR showing the expression of osteogenic differentiation genes and pluripotency-related genes.*$p<0.05$.

Supplemental Table 1. Sequences of primers for real time PCR analysis

Gene Name	Forward primer sequence (5’ to 3’)	Reverse primer sequence (5’ to 3’)
Nanog	GCCCTGATTCTTCTAGCAAT	AGAACACAGTCCGCATCTT
OPN	TCCAAGGAGTATAAGCAGCG GGCCA	CTCTTAGGGTCTAGGACTAGCTT T
OCN	GAGCTGCCCTGCACTGGGTG	TGGCCCCAGACCTCTTCCCG
Runx2	CCGATGGGACCGTGGTT	CAGCAGAGGCATTTCGTAGCT
β-Actin	CGTAAAGACCTCTATGCCAACAT	CGGACTCATCGTACTCCTGCT
Oct4	AGAACCGTGTGAGGTGGAAC	CTCAATGCTAGTCCGCTTTC
Sox2	CCCACCTACAGCATGTCCTA	TGGAGTGGGAGGAAGAGGTA
KDM5C	GAATGTGATGCCTGTGTTGG	CAGGCTGGCTATCAAAAAGC
ESCO1	CCTGTGCCTGTAACTGCTGA	TTTGCTCTTTCCAGGTTGCT
ESCO2	ATCACCACCGATTTCTGGAG	GGCAGGACCAACACAATCTT
HDAC2	TGGCCTTTCTGAGCTGATTT	AGAGGGTCTCTGCCACTGAA
p53	ATGGAGGAGTCACAGTCGGATA	GACTTCTTGTAGATGGCCATGG
CXCR4	TCCGTGGCTGACCTCCTCTT	CAGCTTCCTCGGCCTCTGGC
Dnmt1	GGAAGGTGAGCATCGACGAA	GATCATCCGGAATGACCGAG
ALP	TCCGTGGGTCGGATTCCT	GCCGGCCCAAGAGAGAA

Supplemental Table 2. Sequences of primers for bisulfite sequencing analysis

Promoters	Forward primer sequence (5' to 3')	Reverse primer sequence (5' to 3')
Nanog	TATTATAATGTTTTTGGTGGGTTTG	TCAACCTATCTAAAAACCAACAACTC
Oct4	AGTTTTGAGGTGTTTAGGGATTTAT	CCCCACCAAATAAAAATAAAAAAA

Supplemental Table 3. Sequences of primers for CHIP-PCR analysis

Promoters	Forward primer sequence (5’ to 3’)	Reverse primer sequence (5’ to 3’)
Nanog-1	GCTTGAGGGGGAGGAGTAAG	GCTTGAGGGGGAGGAGTAAG
Nanog-2	GCTTGAGGGGGAGGAGTAAG	GCTTGAGGGGGAGGAGTAAG
Oct4	GTGAGTCGTCCTTCCACCAG	GAGAAGGCGAAGTCTGAAGC
